# Tat-heat shock protein 10 ameliorates age-related phenotypes by facilitating neuronal plasticity and reducing age-related genes in the hippocampus

**DOI:** 10.18632/aging.205182

**Published:** 2023-11-25

**Authors:** Hyo Young Jung, Hyun Jung Kwon, Kyu Ri Hahn, Woosuk Kim, Dae Young Yoo, Yeo Sung Yoon, Dae Won Kim, In Koo Hwang

**Affiliations:** 1 Department of Anatomy and Cell Biology, College of Veterinary Medicine, and Research Institute for Veterinary Science, Seoul National University, Seoul 08826, South Korea; 2 Department of Veterinary Medicine and Institute of Veterinary Science, Chungnam National University, Daejeon 34134, South Korea; 3 Department of Biochemistry and Molecular Biology, Research Institute of Oral Sciences, College of Dentistry, Gangneung-Wonju National University, Gangneung 25457, South Korea; 4 Department of Biomedical Sciences, and Research Institute for Bioscience and Biotechnology, Hallym University, Chuncheon 24252, South Korea; 5 Department of Anatomy, College of Veterinary Medicine, and Veterinary Science Research Institute, Konkuk University, Seoul 05030, South Korea; 6 Department of Anatomy and Convergence Medical Science, Institute of Health Sciences, College of Medicine, Gyeongsang National University, Jinju 52727, South Korea

**Keywords:** heat shock protein 10, aging, hippocampus, memory, neurogenesis, synaptic plasticity

## Abstract

We investigated the effects of heat shock protein 10 (HSP10) protein on memory function, hippocampal neurogenesis, and other related genes/proteins in adult and aged mice. To translocate the HSP10 protein into the hippocampus, the Tat-HSP10 fusion protein was synthesized, and Tat-HSP10, not HSP10, was successfully delivered into the hippocampus based on immunohistochemistry and western blotting. Tat-HSP10 (0.5 or 2.0 mg/kg) or HSP10 (control protein, 2.0 mg/kg) was administered daily to 3- and 21-month-old mice for 3 months, and observed the senescence maker P16 was significantly increased in aged mice and the treatment with Tat-HSP10 significantly decreased P16 expression in the hippocampus of aged mice. In novel object recognition and Morris water maze tests, aged mice demonstrated decreases in exploratory preferences, exploration time, distance moved, number of object contacts, and escape latency compared to adult mice. Treatment with Tat-HSP10 significantly improved exploratory preferences, the number of object contacts, and the time spent swimming in the target quadrant in aged mice but not adults. Administration of Tat-HSP10 increased the number of proliferating cells and differentiated neuroblasts in the dentate gyrus of adult and aged mice compared to controls, as determined by immunohistochemical staining for Ki67 and doublecortin, respectively. Additionally, Tat-HSP10 treatment significantly mitigated the reduction in sirtuin 1 mRNA level, *N*-methyl-D-aspartate receptor 1, and postsynaptic density 95 protein levels in the hippocampus of aged mice. In contrast, Tat-HSP10 treatment significantly increased sirtuin 3 protein levels in both adult and aged mouse hippocampus. These suggest that Tat-HSP10 can potentially reduce hippocampus-related aging phenotypes.

## INTRODUCTION

Aging is characterized by a progressive decline in physiological functions, including antioxidant capacity [1]. With age, the damage accumulates, and the disease susceptibility increases [2]. Various mechanisms of cellular damage with age have been suggested [3–5], among which oxidative stress is most commonly observed [6]. An imbalance between the generation of reactive oxygen species (ROS) and their elimination can cause the accumulation of ROS in cells, which oxidize large molecules such as DNA, phospholipid membranes, and mitochondria, ultimately resulting in tissue damage [7, 8]. In particular, the brain is susceptible to cell damage induced by ROS with aging because it utilizes a large amount of oxygen, and neurons have a high content of unsaturated fatty acids [9]. Therefore, ROS accumulation is closely linked to neurodegenerative diseases [10–12] as well as cognitive disorders and dementia [13].

The hippocampus, located in the medial temporal lobe, plays a major role in consolidating episodic memory and spatial learning [14]. The hippocampus is highly vulnerable to age-related damage [15], which causes memory impairment and is considered an indicator of aging phenotypes in rodents [16]. In addition, the hippocampal dentate gyrus is one of the major neurogenic regions throughout life, and newly-generated neurons in the subgranular zone of the dentate gyrus migrate to the granule cell layer of the dentate gyrus [17]. Several studies have demonstrated a relationship between neurogenesis and memory. Adult hippocampal neurogenesis dramatically decreases with the aging process [18, 19], and the enhancement of neurogenesis in aged mice improves memory deficits [20, 21].

Heat shock proteins (HSPs), known as molecular chaperones, are required for protein folding, transportation, and maturation under physiological conditions [22]. However, when ROS formation increases, HSPs are rapidly synthesized and involved in stabilizing the denatured proteins [23, 24]. HSPs are classified depending on their molecular size; HSP60/HSP10 plays an important role in modulating mitochondrial function [25] and preventing the aggregation of damaged proteins [26]. HSP10 expression levels decreased in the striatum of mice and the putamen of humans with Parkinson’s disease [27] and in the hypothalamus of diabetic animals [28]. In contrast, HSP60 and HSP10 mRNA levels significantly increased after ischemic damage [29]. Overexpression of HSP10 prevented contraction-induced damage in muscles [30], whereas mutations in HSP10 are associated with neurological and developmental disorders [31].

Much evidence shows the biomarkers associated with senescence. Particularly, P16, P21, and senescence-associated β-galactosidase are considered the typical biomarkers [32]. In addition, sirtuins (Sirts) have histone deacetylase activity in a nicotinamide adenine dinucleotide-dependent manner and play important roles in homeostasis. Sirts are closely related to brain aging and longevity pathway, including forkhead box O (FOXO) signaling. Furthermore, the reduction of cognitive function in aging can be explained by changes in synaptic plasticity [33] by regulating *N*-methyl-D-aspartate receptor 1 (NMDAR1). In addition, postsynaptic density 95 (PSD95) regulates NMDAR-dependent synaptic plasticity [34]. Vesicular glutamate transport 2 (VGLUT2) is a valuable marker for canonical glutamatergic neurons, although it is expressed in nerve endings [35]. Although HSP10 has positive effects on various diseases, no studies have been conducted on the HSP10 effects on hippocampal function in aged animals. In the present study, we synthesized a Tat-HSP10 fusion protein to cross the HSP10 to the brain parenchyma and observed the effects of the Tat-HSP10 fusion protein on memory functions via novel object recognition and Morris water maze test in adult and aged mice. In addition, we investigated cell proliferation/differentiation, the hippocampal mRNA expression of aging-related genes, such as Sirt1, Sirt2, and FoxO1; and proteins related to synaptic plasticity, such as NMDAR1, PSD95, and VGLUT2, as well as aging-related markers such as P16 and Sirt3.

## RESULTS

### Confirmation of delivery of Tat-HSP10 and HSP10 proteins and their effects on mitochondrial proteins

**Figure 1:**
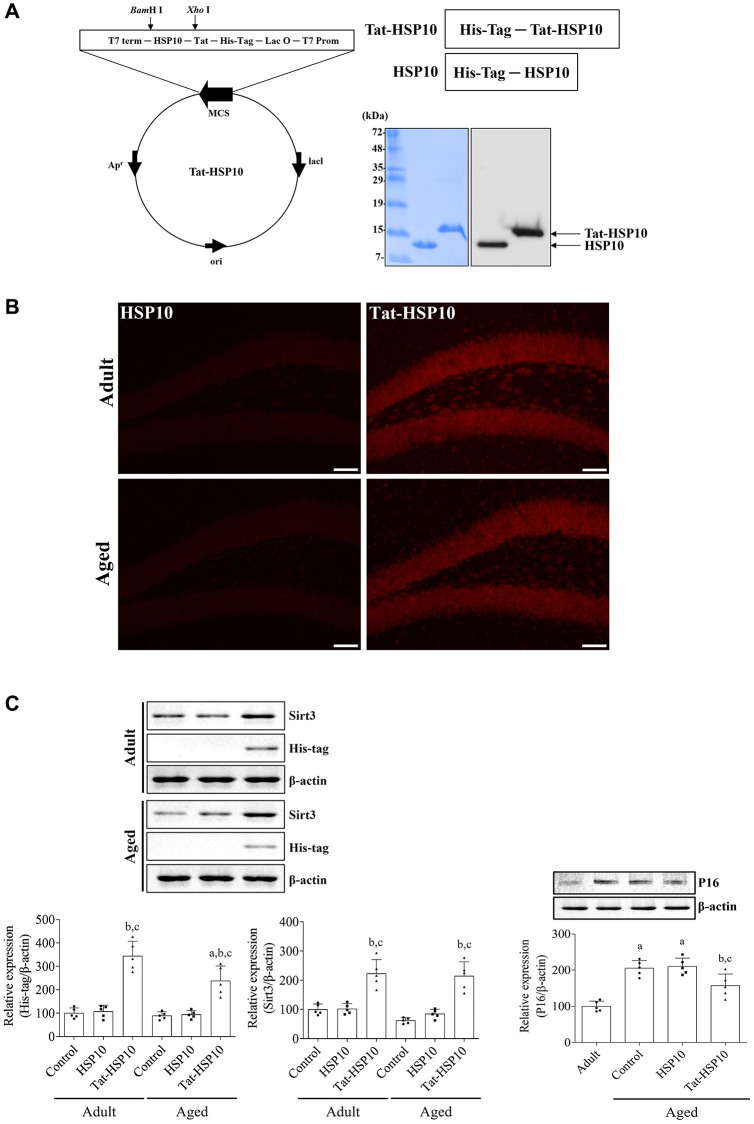
**Synthesis of Tat-HSP10 and HSP10 proteins, their delivery into the hippocampus, and their effects on mitochondrial marker proteins.** (**A**) Schematic diagrams of vectors encoding Tat-HSP10 and HSP10 are shown, and the expression is visualized and confirmed using Coomassie Brilliant Blue staining and western blotting for His-Tag. Clear bands are detected. Tat-HSP10 protein is detected at a slightly higher molecular weight compared to HSP10. (**B**) Delivery of Tat-HSP10 and HSP10 is visualized by immunohistochemical staining for His-Tag. His-Tag immunoreactive structures are abundantly detected in the Tat-HSP10-treated group, not in the HSP10-treated group. HSP, Heat shock protein; His, histidine. (**C**) Protein expressions such as His-Tag, Sirt3, and P16 are assessed by western blotting. His-Tag protein is highly expressed in the Tat-HSP10-treated group of adult and aged mice, but the protein level is significantly low in the aged group compared to that in the adult group. Sirt3 protein levels are increased in Tat-HSP10 treated groups of both the adult and aged groups. P16 levels show significantly higher levels in aged mice than in adult ones, and the protein level is significantly decreased in the aged group compared to the adult group. Data are represented as the mean ± SD (*n* = 5 each group; analyzed by one-way or two-way ANOVA test followed by Tukey’s post hoc test, ^a^*P* < 0.05, vs. adult group; ^b^*P* < 0.05, vs. control group; ^c^*P* < 0.05, vs. HSP10-treated group). Abbreviations: HSP: Heat shock protein; Sirt: sirtuin; ANOVA: Analysis of Variance; SD: standard deviation.

After synthesis, overexpression, and purification with plasmids encoding Tat-HSP10 and HSP10, the final product was confirmed by western blotting for His-Tag because 6x histidine residues were inserted into the plasmid, as shown in Fig. 1. Tat-HSP10 and HSP10 proteins were observed using Coomassie Brilliant Blue staining and electrochemiluminescence membranes. The Tat-HSP10 band was detected at a slightly higher molecular weight than that of HSP10 because of the differences in molecular weight with the Tat peptide (1.6 kDa) (Figure 1A).

To confirm the delivery of proteins to the mouse hippocampus, His-Tag immunohistochemical staining was performed 1 h after protein treatment. In the HSP10-treated group, little His-Tag immunoreactive structures were found in the dentate gyrus of adult (6-month-old) and aged (24-month-old) mice. In contrast, His-Tag immunoreactivity in the Tat-HSP10-treated group was observed in the granule cell layer and the polymorphic layer of the dentate gyrus. More His-Tag immunoreactive structures were found in the dentate gyrus of compared HSP10-treated adult group (Figure 1B). Based on the western blotting, His-Tag protein levels were significantly increased in adult (344.1% of adult control, *p* < 0.0001) and aged (238.1% of adult control, *p* < 0.0001) groups compared respective control group. In the Tat-HSP10-treated group, the His-Tag protein level was significantly lower (*p* = 0.0037) in the aged group than in the adult group (Figure 1C).

Tat-HSP10-induced mitochondrial function was assessed by western blotting for Sirt3. The Sirt3 protein level of aged mice was decreased in the hippocampus to 61.8% compared to adult mice, although statistical significance was not detected (*p* = 0.3761). However, treatment with Tat-HSP10 showed significant increases in Sirt3 protein levels in both adult and aged mice (*p* < 0.001) to 223.5% and 215.1% of the adult control group compared to respectively control and HSP10-treated group (Figure 1C).

P16, a classical senescence marker, protein levels in aged mice were significantly increased (*p* < 0.0001) in the hippocampus to 205.4% that of adult mice. In the HSP10-treated group, P16 protein levels did not show any significant changes (*p* = 0.9870) in the hippocampus of aged mice compared to that in the aged control group. However, in the Tat-HSP10-treated group, P16 protein levels were significantly increased to 157.0% of the control group compared to the aged control (*p* = 0.0232) or HSP10-treated (*p* = 0.0119) groups (Figure 1C).

### Tat-HSP10 enhances age-related cognitive impairments

Adult mice showed significantly increased exploratory preferences for novel objects but showed no significant changes in novel object preference after HSP10 or Tat-HSP10 treatment. Whereas aged mice did not show any significant preference for novel objects, and a similar pattern of preference was observed after treatment with HSP10. However, Tat-HSP10 administration significantly increased the preference for novel objects compared to familiar objects in aged mice. Additionally, the novel object preference was significantly increased, and familiar object preference decreased after Tat-HSP10 treatment in aged mice compared to the control group.

**Figure 2:**
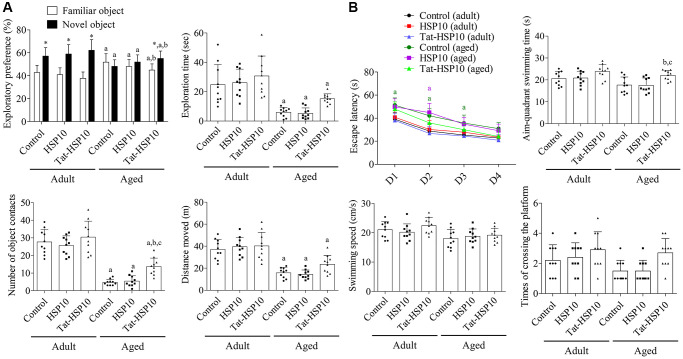
Effects of Tat-HSP10 and HSP10 on novel object recognition (**A**) and Morris water maze task (**B**) in adult and aged mice. (**A**) Exploratory preference (familiar vs. new object), total exploration time, number of contacts with a novel object during the testing trial, and distance moved were analyzed in control, HSP10-, and Tat-HSP10-treated groups of adult and aged mice. (**B**) Escape latency was analyzed in four consecutive navigation tests. The time consumed in the aim quadrant, swimming speed, and platform crossing times were measured on the next navigation test in the control, HSP10-, and Tat-HSP10-treated groups of adult and aged mice. Data are represented as the mean ± SD (*n* = 10 each group; analyzed by one-way or two-way ANOVA tests followed by Tukey’s post hoc test, ^*^P < 0.05, vs. the familiar object; ^a^*P* < 0.05, vs. adult group; ^b^*P* < 0.05, vs. control group; ^c^*P* < 0.05, vs. HSP10-treated group). Abbreviations: HSP: Heat shock protein; ANOVA: Analysis of Variance; SD: standard deviation.

Aged group mice spent significantly less time exploring novel objects than those in the adult group. In addition, the number of object contacts and travel distance were significantly lower in the aged group than in the adult group. HSP10 treatment showed similar levels of exploration time, number of object contacts, and travel distance compared to those in the control group of adult and aged mice. However, treatment with Tat-HSP10 ameliorated the reduction in exploration time, number of object contacts, and travel distance in the adult and aged groups compared to their respective control groups. In particular, Tat-HSP10 treatment in aged mice significantly increased the number of object contacts (291.5% of the control group compared to the aged control group (*p* = 0.0113) or HSP10-treated (*p* = 0.0242) group (Figure 2A).

In the testing trial, aged mice in the control group showed significantly longer escape latency on Morris water maze test day 1 (*p* = 0.0028), 2 (*p* = 0.0004), and 3 (*p* = 0.0330) compared to adult mice. In the HSP10-treated group, the escape latency was significantly decreased in aged mice only on day 2 (*p* < 0.0001) compared to that in the adult group. Meanwhile, escape latency was not significantly changed after Tat-HSP10 treatment between the adult and aged groups. The administration of HSP10 or Tat-HSP10 showed no significant differences in escape latency in both adult and aged groups. In the probing trial, the time spent swimming in the target quadrant was significantly increased in the aged Tat-HSP10-treated group compared to the aged control group (*p* = 0.0302) or the HSP10-treated (*p* = 0.0255) group. Swimming speed tended to decrease in the aged group, and the platform crossing times were increased in the Tat-HSP10-treated groups of both the adult and aged groups. However, statistical significance was not detected among groups (Figure 2B).

### Tat-HSP10 increases cell proliferation and neuroblast differentiation in the dentate gyrus of adult and aged mice

**Figure 3:**
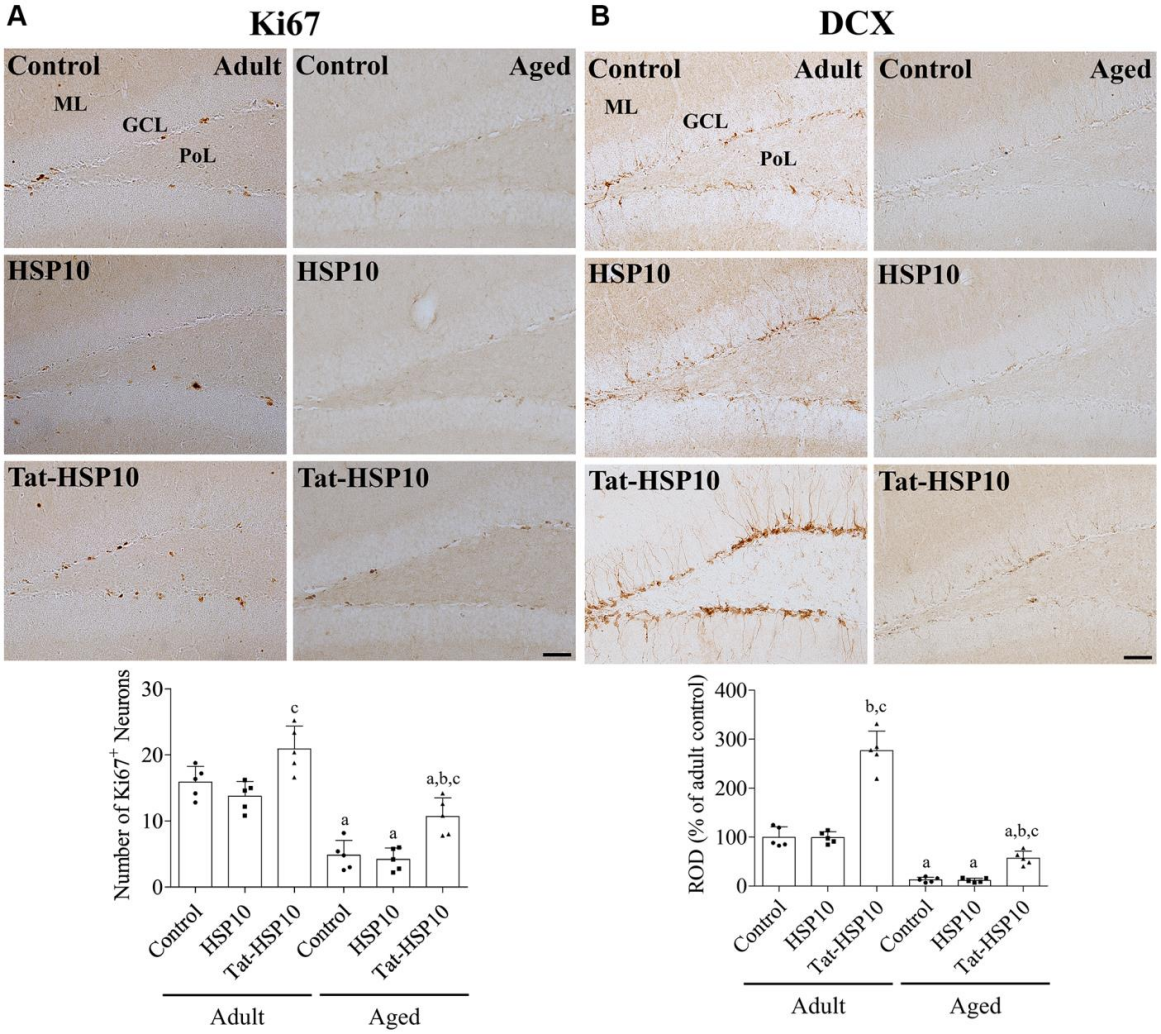
**Effects of Tat-HSP10 and HSP10 on cell proliferation and neuroblast differentiation in adult and aged mice.** Immunohistochemical staining for (**A**) Ki67 and (**B**) DCX are conducted to visualize the proliferating cells and differentiated neuroblasts in the dentate gyrus, respectively, in the control, HSP10-, and Tat-HSP10-treated groups of adult and aged mice. Scale bar = 50 μm. Immunohistochemical staining is quantified by counting the Ki67-immunoreactive nuclei in the subgranular zone and measuring the immunodensity of DCX-immunoreactive neuroblasts in the whole dentate gyrus. The immunodensity of DCX is normalized into percentile value vs. the control group of adult mice. Data are represented as the mean ± SD (*n* = 5 each group; analyzed by two-way ANOVA test followed by Tukey’s post hoc test, ^a^*P* < 0.05, vs. adult group; ^b^*P* < 0.05, vs. control group; ^c^*P* < 0.05, vs. HSP10-treated group). Abbreviations: HSP: Heat shock protein; ANOVA: Analysis of Variance; SD: standard deviation; DCX: doublecortin.

In all groups, Ki67 immunoreactivity was found in the nucleus, located in the subgranular zone of the dentate gyrus. In contrast, doublecortin (DCX) immunoreactivity in all groups was detected in the cytoplasm of the subgranular zone, granule cell layer, and dendrites in the molecular layer of the dentate gyrus. DCX immunoreactivity and the number of Ki67-immunoreactive nuclei were significantly lower in aged mice than in adult mice. Treatment with HSP10 showed slightly lower numbers of Ki67-immunoreactive nuclei or similar DCX immunoreactivity compared to the control group of adult and aged mice. Whereas administration of Tat-HSP10 significantly increased Ki67 immunoreactive nuclei (131.5% (*p* = 0.0464) and 222.5% (*p* = 0.0134) in adult and aged mice, respectively) and DCX immunoreactivity (276.5% (*p* < 0.0001) and 444.4% (*p* = 0.0202) in adult and aged mice, respectively) compared to the control groups (Figure 3).

### Tat-HSP10 improves the Sirt1-FoxO1 pathway and synaptic plasticity in aged mice

**Figure 4:**
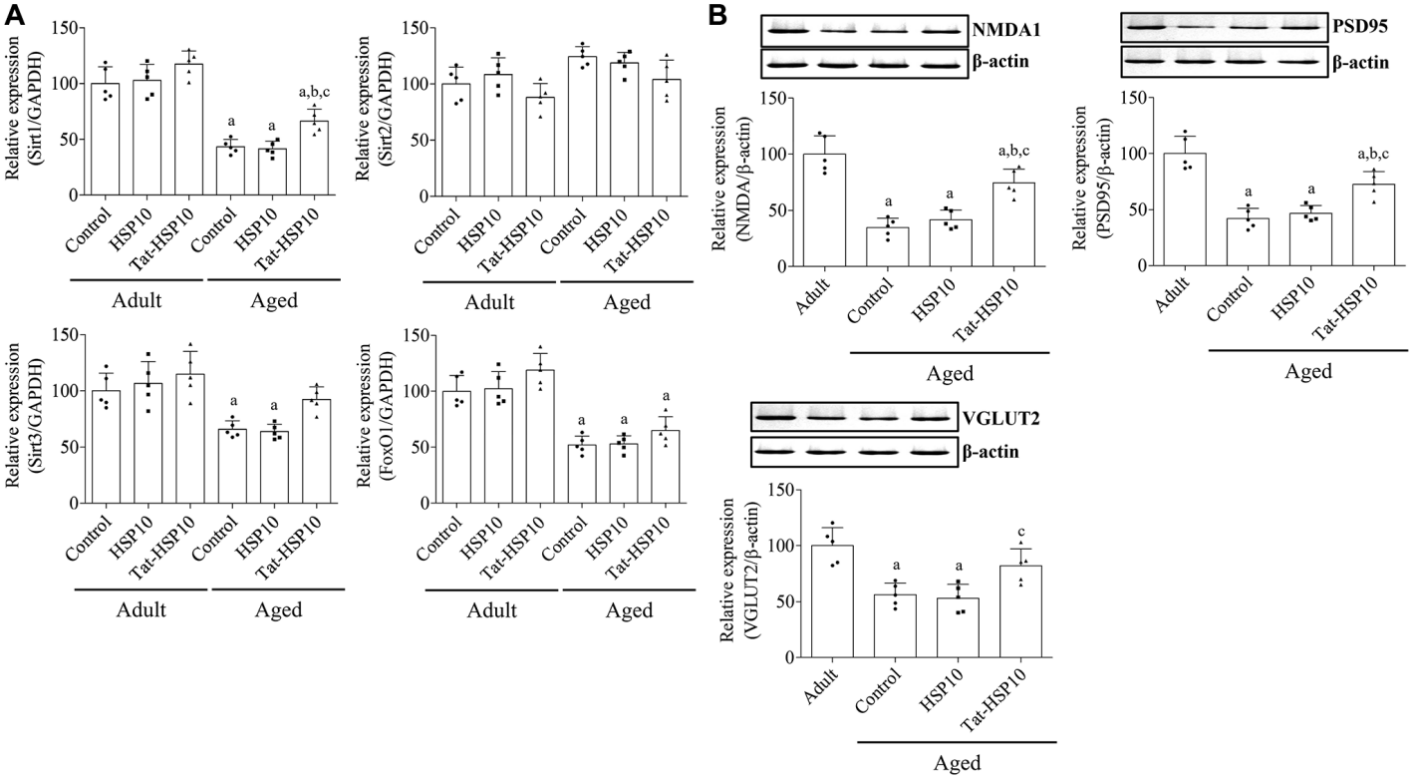
**Effects of Tat-HSP10 and HSP10 on the expression of aging-related genes and synaptic plasticity-related proteins in the hippocampus.** (**A**) Real-time PCR is performed to detect Sirt1, Sirt2, and FoxO1 mRNA levels in the control, HSP10-, and Tat-HSP10-treated groups of adult and aged mice. In addition, (**B**) NMDAR1, PSD95, and VGLUT2 protein levels are detected using western blotting in aged mice. Data from western blotting are quantified based on the immunodensity of each band. Data are represented as the mean ± SD (*n* = 5 each group; analyzed by two-way ANOVA test followed by Tukey’s post hoc test, ^a^*P* < 0.05, vs. adult group; ^b^*P* < 0.05, vs. control group; ^c^*P* < 0.05, vs. HSP10-treated group). Abbreviations: HSP: Heat shock protein; ANOVA: Analysis of Variance; SD: standard deviation; Sirt1: sirtuin 1; Sirt2: sirtuin 2; FoxO1: forkhead box O1; NMDAR1: *N*-methyl-D-aspartate receptor 1; PSD95: postsynaptic density 95; VGLUT2: vesicular glutamate transport 2.

Sirt1 and FoxO1 mRNA expressions were significantly lower (*p* < 0.0001) in aged mice than in adult mice, whereas Sirt2 expression was not significantly different (*p* = 0.0716) between adult and aged mice. HSP10 treatment did not affect the mRNA expression of Sirt1, Sirt2, or FoxO1 in adult and aged mice. However, the administration of Tat-HSP10 showed a tendency to increase Sirt1 and FoxO1 expression only in aged mice compared to that in the control group (Figure 4A). In particular, Sirt1 mRNA expression in aged mice was significantly higher (*p* = 0.0390) than that in the control group (149.3%).

In aged mice, NMDAR1, PSD95, and VGLUT2 expressions were significantly decreased to 34.7% (*p* < 0.0001), 42.2% (*p* < 0.0001), and 56.2% (*p* = 0.0006), respectively, compared to adult mice. Administration of HSP10 did not result in any significant changes in protein expression compared to that in the control group. However, treatment with Tat-HSP10 significantly alleviated NMDAR1 and PSD95 expressions to 74.4% (*p* = 0.0004) and 72.7% (*p* = 0.0026), respectively, in adult mice compared to those in aged mice (Figure 4B).

## DISCUSSION

Mitochondrial dysfunction is a major cellular change observed in the hippocampus during aging [36]. HSP60 and HSP10, mitochondrial chaperones, facilitate protein folding and are located on chromosome 2, controlled by a bidirectional promoter. Targeting HSP10 causes a significant reduction in mitochondrial activity and ATP production [28]. A recent study demonstrated a higher abundance of HSP10 in the hippocampus than HSP60 [37] despite its head-to-head gene location. In the present study, we investigated the effects of HSP10 on hippocampal function in both adult and aged mice. To cross the blood-brain barrier, we synthesized the Tat-HSP10 fusion protein because it can translocate a conjugated protein across the cell membrane and blood-brain barrier [38, 39]. In previous studies, Tat-conjugated protein translocation across the blood-brain barrier has been reported in the mouse hippocampus [40, 41]. In the present study, we observed the expression of Tat-HSP10 and HSP10 proteins after synthesis, overexpression, and purification. In addition, we observed the conjugation of the Tat peptide and HSP10 by western blotting, which was confirmed by the differences in molecular weight between Tat-HSP10 and HSP10.

Furthermore, we confirmed the delivery of proteins into mouse hippocampus 1 h after protein treatment by immunohistochemical staining and western blotting for His-Tag. This result suggests that Tat-HSP10 protein is quickly delivered into the mouse hippocampal dentate gyrus, including granule cells and interneurons in the polymorphic layer of the dentate gyrus. However, in the present study, we observed significantly lower levels of His-Tag expression in the hippocampus of aged mice than in adult mice. However, significantly higher protein expression was found in the hippocampus of aged mice than in the control group. This result suggests that the transduction efficacy of Tat-HSP10 decreased in aging animals.

In the present study, we excluded the Tat peptide group because several studies demonstrated that Tat peptide produced memory deficits in terms of novel object recognition and the Morris water maze test [42]. In addition, Tat peptide decreased the proliferation, migration, and differentiation of neural stem cells [43, 44]. We screened the markers such as Sirt3 and P16 to observe age-related mitochondrial function and senescence after Tat-HSP10 treatment. Sirt3, an important deacetylase in mitochondria, plays a crucial role in mitochondrial quality control [45]. Overexpression of Sirt3 attenuated anesthesia-induced learning and memory dysfunction [46] and ameliorated diabetes-induced cognitive impairment and mitochondrial dysfunction [47]. Reduced Sirt3 expression causes hyperacetylation of mitochondrial proteins linked with neuronal excitotoxicity and cell death [45]. The depletion of Sirt3 in mice showed cognitive impairments during the probe trial in the Morris water maze test [48].

In the present study, we observed significant increases in Sirt3 levels after Tat-HSP10 treatment, suggesting that mitochondrial function may be enhanced by treatment with Tat-HSP10 in the hippocampus of adult and aged mice. P16 inhibits the G_0_/G_1_ cell cycle via the enhancement of P21 stability, and the expression was enhanced in the hippocampus of D-galactose-induced aged mice [49]. In addition, the overexpression of P16 facilitates senescence and negatively affects lifespan [50]. Consistent with previous studies [51, 52], P16 protein expression was significantly increased in the hippocampus of aged mice compared to adult mice. Treatment with Tat-HSP10 significantly ameliorated the increase of P16 protein in the aged hippocampus, suggesting that Tat-HSP10 can be a candidate for anti-aging therapeutics.

Next, we examined the effect of Tat-HSP10 on cognition memory using novel object recognition and the Morris water maze test to elucidate the effects of Tat-HSP10 against aging-induced cognitive impairments using two independent memory tests. Adult mice showed a higher exploratory preference for novel objects than familiar objects, but aged mice showed lower exploratory activity based on exploratory time, the number of object contacts, and the distance moved. In addition, aged mice had less than 50% preference, which indicates memory impairment [53, 54]. In this study, the administration of Tat-HSP10 demonstrated more than 50% preference for novel objects, and we observed statistically significant differences in preference. In addition, we found that the number of object contacts was significantly higher in the Tat-HSP10-treated aged mice. In the Morris water maze test, we found significant decreases in escape latency during the testing period and significant increases in the time spent swimming in the target quadrant during the probing trial. Collectively, Tat-HSP10 enhances cognitive function in aged (not adult) mice.

We also observed cell proliferation and neuroblast differentiation in the dentate gyrus because adult neurogenesis is closely related to learning, memory, mood, and cognitive flexibility [55, 56]. Aged mice had significantly fewer Ki67-immunoreactive proliferating cells and fewer DCX immunoreactive differentiated neuroblasts compared to adult mice. This result is consistent with previous studies showing that adult neurogenesis is inversely correlated with age [18, 19]. In contrast, increased adult neurogenesis rejuvenates memory circuits, learning, and memory [20, 21]. We observed that the administration of Tat-HSP10 significantly increased the number of proliferating cells in aged mice and differentiated neuroblasts in both adult and aged mice. This result suggests that Tat-HSP10 ameliorates cognitive impairment in aged mice, which may be associated with the enhancement of hippocampal neurogenesis. Antidepressants, such as venlafaxine or fluoxetine, significantly increase adult hippocampal neurogenesis.

Interestingly, the induction of HSP10 expression has been reported after antidepressant treatment in the rat hippocampus [57]. In peripheral organs, HSP10 overexpression prevents age-related atrophy of skeletal muscles in old mice by reducing the accumulation of protein carbonyls [30]. Additionally, treatment with exogenous HSP10 prolonged survival time after skin grafting [58] and reduced tissue damage from myocardial infarction [59, 60].

To elucidate the effects of Tat-HSP10 on age-related gene expression, we conducted real-time polymerase chain reaction (PCR) analysis for FoxO1, Sirt1, and Sirt2 in the hippocampus because FoxO1 and Sirt1 are abundantly expressed in the hippocampus and decrease with the aging process [61, 62]. We observed Sirt2 mRNA levels in the hippocampus, which are not directly associated with hippocampal senescence [63]. Consistent with previous studies [61, 62, 64], FoxO1 and Sirt1 mRNA levels were significantly decreased in aged mice, but Sirt2 mRNA levels were not significantly different between the groups. Treatment with Tat-HSP10 significantly alleviated this reduction in Sirt1 mRNA levels in aged mice. We also performed western blotting for NMDAR1, PSD95, and VGLUT2, which are hippocampal markers of synaptic plasticity [65–67]. As reported in previous studies, NMDAR1, PSD95, and VGLUT2 protein expressions were significantly decreased in the hippocampus during aging [68–70]. Treatment with Tat-HSP10 significantly ameliorated the reduction in NMDAR1 and PSD95 levels in the hippocampi of aged mice. A recent study in mice demonstrated that Sirt1 knockdown in the hippocampus reduced PSD95 levels, which causes spatial learning and memory deficits [71].

In conclusion, Tat-HSP10 is efficiently delivered into the hippocampus of adult and aged mice, but the efficacy was higher in the adult group than in the aged group. In addition, the delivered Tat-HSP10 protein increases a mitochondrial functional protein such as Sirt3 and ameliorates classical senescence markers such as P16 in both adult and aged mice. In addition, Tat-HSP10 ameliorated cognitive deficits in aged mice by increasing the number of proliferating cells and differentiated neuroblasts, probably by alleviating the aging-induced reduction in Sirt1, NMDAR1, and PSD95 in the hippocampus. Our results suggest that Tat-HSP10 treatment facilitates mitochondrial function, and Tat-HSP10 supplementation ameliorates the aging phenotypes in the mouse hippocampus.

## MATERIALS AND METHODS

### Synthesis of Tat-HSP10 and its control protein

Two oligonucleotides encoding the basic domain of HIV-1 Tat (amino acids 49–57) were synthesized, annealed, and ligated into a *Nde*I-*Xho*I-digested pET15b vector with 6x histidine, as described previously [72, 73]. Sense and antisense primers for human HSP10 cDNA (Takara Bio Inc., Shiga, Japan) were synthesized and amplified using PCR. The digestion of PCR products was performed using *Xho*I and *Bam*HI, and the resulting products were ligated into a TA cloning vector (Promega, Madison, WI, USA) with a Tat vector. Plasmids encoding Tat-HSP10 and its control (HSP10) were transformed into *E. coli* BL21 (DE3) and colonies were selected on Luria-Bertani medium.

Induction of overexpressed proteins was performed by adding 0.5 mM isopropyl-β-D-thiogalactoside (IPTG; Duchefa, Haarlem, The Netherlands). Five hours after treatment with IPTG, cells were harvested and lysed; the proteins were purified using a Ni^2+^-nitrilotriacetic acid Sepharose column (Qiagen, Inc., Valencia, CA, USA); and desalted by PD-10 desalting column chromatography (GE Healthcare, Piscataway, NJ, USA). Expression of Tat-HSP10 and HSP10 proteins was confirmed by western blotting for His-Tag to detect 6x histidine as described previously [40, 72].

### Experimental animals and treatment with Tat-HSP10 and HSP10

Male C57BL/6 mice (5 weeks old; Japan SLC Inc., Shizuoka, Japan) were housed in the specific pathogen-free facility of Seoul National University College of Veterinary Medicine. The experimental protocols for the animal study were approved by the Institutional Animal Care and Use Committee of the Seoul National University (SNU-160929-5-2). At 3 and 21 months of age, mice were intraperitoneally injected with 1 mg/kg Tat-HSP10 or HSP10 once a day for 3 months. To confirm the delivery of proteins into the hippocampus, 3-month-old mice received intraperitoneal injection of 1 mg/kg Tat-HSP10 or HSP10 and the animals were sacrificed 1 h after protein treatment.

### Novel object recognition test

After 13 weeks of protein treatment, a novel object recognition test was performed to evaluate the recognition memory in mice. Mice were freed in a black acrylic box (25 cm × 25 cm × 25 cm) for 2 min adaptation, as described previously [74]. For the training trial, two identical objects were placed in the opposite corners 24 h after adaption (1 h after protein treatment), and mice (*n* = 10 in each group) were allowed to explore the box for 2 min. After 1 h of the training trial, one object was replaced with a new one, and the mice were freed to explore the familiar and new objects for 2 min. Exploratory preference was determined with percentile value by dividing the total exploratory time by the time to explore the object. The number of object contacts, exploration time, and distance traveled were calculated in all groups when the animals were approached within 2 cm of the new object.

### Morris water maze test

After 13 weeks of protein treatment, the Morris water maze test was performed to evaluate the spatial learning memory in mice. For the water maze test, animals were released to find the platform in a circular pool (100 cm in diameter) filled with powdered milk-dissolved water (24–25°C). The escape platform (7 cm in diameter) was submerged within 5 mm of the water’s surface. The navigation test was conducted for 60 s (four trials per day for four consecutive days), and the time consumed to find the hidden platform (escape latency) was measured using the Videomex tracking system (Columbus Instruments, Columbus, OH, USA). The next day, mice were freed into the pool at a random quadrant, and a spatial probe test was performed to measure the time to cross the platform, swimming speed, and time spent swimming in the target quadrant within 60 s using the Videomex tracking system (Columbus Instruments).

### Immunohistochemistry

Immediately after the novel object recognition test or 1 h after protein treatment (for confirmation of protein delivery), the mice (*n* = 5 in each group) were anesthetized with a mixture of xylazine (10 mg/kg; Bayer Korea, Seoul, South Korea) and alfaxalone (75 mg/kg; Careside, Seongnam, South Korea). Transcardiac perfusion was performed via the left ventricle using physiological saline and 4% paraformaldehyde solution. Hippocampal sections (30 μm thickness) located between 1.82 and 2.30 mm caudal to the bregma [75] were obtained using a sliding microtome with a freezing station (HM430; *Thermo Scientific*, Waltham, MA, USA) after post-fixation and cryoprotection with same fixative and 30% sucrose solution, respectively. Three sections (120 μm apart from each other) were selected, and immunohistochemical staining was conducted, as described in the previous studies [40, 72, 74]. Briefly, rabbit anti-His-Tag (1:1000, Cat#NBP2-61482, Novus Biologicals, Centennial, CO, USA), rabbit anti-Ki67 antibody (1:1000, Cat# ab15580, Abcam, Cambridge, UK), rabbit anti- DCX antibody (1:5000, Cat# ab18723, Abcam), biotinylated goat anti-rabbit IgG (1:200, Cat# BA-1000, Vector Lab., Burlingame, CA, USA), and the VECTASTAIN Elite^®^ ABC system (Vector Laboratories, Burlingame) were used except for His-Tag. Immunoreactive signals were visualized using 3,3′-diaminobenzidine tetrachloride (Sigma, St. Louis, MO, USA). For His-Tag, the sections were incubated with Cy3-conjugated anti-rabbit IgG (1:100; Jackson ImmunoResearch Laboratories Inc., West Grove, PA, USA).

### Western blot and quantitative PCR

Immediately after the novel object recognition test, mice (*n* = 5 in each group) were anesthetized. Left and right hippocampal tissues were isolated for western blotting and quantitative PCR, respectively, as described previously [76, 77]. Sodium dodecyl sulfate-polyacrylamide gel electrophoresis and nitrocellulose membranes (Pall Crop, East Hills, NY, USA) were used. Rabbit anti-NMDAR1 (1:1000, Cat# 5704, Cell Signaling, Danvers, MA, USA), rabbit anti-PSD95 (1:1000, Cat# ab18258, Abcam), rabbit anti-VGLUT2 (1:5000, Cat# ab79157, Abcam), mouse anti-P16 (1:500; Cat#sc-377412, Santa Cruz Biotechnology, Santa Cruz, CA, USA), rabbit anti-His-Tag (1:1000, Cat#NBP2-61482, Novus Biologicals), rabbit anti-Sirt3 (1:1000; Cat#ab189860, Abcam), and peroxidase-conjugated goat anti-rabbit IgG (Cat# PI-1000-1, Vector Lab.) or peroxidase-conjugated horse anti-mouse IgG (Cat# PI-2000-1, Vector Lab.) were used to identify respective proteins. For quantitative real-time PCR, specific forward and reverse primers were synthesized as follows: Foxo1 mRNA forward primer (5′-ACATTTCGTCCTCGAACCAGCTCA-3′) and reverse primer (5′-ATTTCAGACAGACTGGGCAGCGTA-3′), Sirt1 mRNA forward primer (5′-CGGCTACCGAGGTCCATATAC-3′) and reverse primer (5′-CAGCTCAGGTGGAGGAATTGT-3′), and Sirt2 mRNA forward primer (5′-GAGCCGGACCGATTCAGAC-3′) and reverse primer (5′-AGACGCTCCTTTTGGGAACC-3′).

### Statistical analysis

All experiments were conducted simultaneously, and the data were analyzed by two independent observers. Data are presented as mean ± standard deviation (SD). The effects of Tat-HSP10 or HSP10 depending on aging were analyzed by a two-way analysis of variance (ANOVA) followed by Tukey’s multiple comparisons test using GraphPad Prism 9.5 software (GraphPad Software, Inc., La Jolla, CA, USA).
